# Alpine steppe degradation weakens ecosystem multifunctionality through the decline in climax dominant species on the Qinghai-Tibetan plateau

**DOI:** 10.3389/fpls.2025.1650352

**Published:** 2025-10-06

**Authors:** Xiao-Yun Wang, Yuan-Ming Xiao, Wen-Ying Wang, Xin-Yu Yang, Guo-Ying Zhou

**Affiliations:** ^1^ Academy of Animal and Veterinary Science, Qinghai University, Xining, China; ^2^ Northwest Institute of Plateau Biology, Chinese Academy of Sciences, Xining, China

**Keywords:** grassland degradation, ecosystem multifunctionality, plant community diversity, dominant species, alpine steppe

## Abstract

**Introduction:**

The diversity and dominant species of plant communities are vital for maintaining grassland ecosystem multifunctionality. However, grassland degradation can disrupt plant community diversity, dominant species, and their linkages with ecosystem multifunctionality.

**Methods:**

We studied the alpine steppe in the Qinghai Lake Basin, conducting plant community surveys and sampling at 15 sites across four degradation gradients (non-degraded, lightly degraded, moderately degraded, and severely degraded). This study investigated the relationships among plant community diversity, dominance of dominant species, and ecosystem multifunctionality (derived via factor analysis from 11 indicators: aboveground biomass, belowground biomass, plant height, coverage, TN, TP, AN, AP, SOC, SM, and pH) in the context of alpine steppe degradation.

**Results:**

The results revealed that plant community diversity-measured using the Shannon-Wiener index, Simpson index, species richness, and Pielou evenness index—followed a unimodal trend with increasing degradation, peaking at moderate degradation levels. Meanwhile, belowground biomass, soil nutrient and moisture content declined significantly with degradation severity. Regression analysis revealed that alpine steppe ecosystem multifunctionality followed a binomial rather than linear relationship with plant diversity and dominance of dominant species across degradation gradients. In non-degraded and moderately degraded alpine steppe, ecosystem multifunctionality responded significantly to Shannon-Wiener index, Simpson index, and species richness, but not to Pielou evenness. During the degradation process of alpine steppe, the linear mixed model results demonstrated that the dominance of dominant species significantly influenced ecosystem multifunctionality.

**Discussion:**

Consequently, in the ecological restoration of degraded alpine steppe, precedence should be accorded to the establishment of dominant species and the enhancement of soil conditions, subsequently followed by the optimization of plant community diversity.

## 1 Introduction

Ecosystem multifunctionality refers to the simultaneous provision of multiple ecosystem services and functions, such as primary production, climate regulation, and water resource protection (Manning et al., 2018; Qiu et al., 2021; Zavaleta et al., 2010; Zhang et al., 2024). As key components of terrestrial ecosystems, grasslands sustain critical functions that directly support human well-being (Dong et al., 2020; Zhao et al., 2020). Plants form the foundation of these ecosystems, serving as primary producers and a major driver of multifunctionality (Wang et al., 2023a; Zhu et al., 2023). Plant diversity plays a pivotal role in sustaining grassland multifunctionality (Qin et al., 2025), with extensive evidence showing that reduced diversity impairs key ecosystem processes, including net primary productivity, nutrient cycling, and stability (López-Rojo et al., 2019; Maestre et al., 2012). Thus, understanding the link between plant diversity and ecosystem multifunctionality has become a central focus in ecology. However, while plant diversity significantly influences multifunctionality, an exclusive focus on diversity metrics often neglects the substantial role of dominant species in ecosystem functioning (Chen et al., 2023; Garnier et al., 2004). Dominant species—those with high relative abundance or biomass-exert strong control over ecosystem processes (Avolio et al., 2019; Zhang et al., 2025). Experimental studies demonstrate that their removal can drastically alter community diversity and ecosystem function (McCain et al., 2010; Wardle et al., 1999). Moreover, dominant species mediate environmental change impacts on plant communities and enhance the temporal stability of ecosystem functions (Grman et al., 2010; Hoover et al., 2014; Hou et al., 2023; Sasaki and Lauenroth, 2011). Despite their importance, the relative contributions and interactions between plant diversity and dominant species in driving ecosystem services remain poorly understood.

Alpine grasslands constitute a vital component of the Tibetan Plateau’s ecological system, playing crucial roles in maintaining ecological stability and supporting human livelihoods (Cheng et al., 2023; Qian et al., 2021). However, these ecosystems are currently experiencing severe degradation (Liu et al., 2024; Wang et al., 2019; Yang et al., 2019). This degradation leads to deterioration in vegetation and soil quality, shifts in dominant species composition, and reduced biodiversity and ecosystem services (Wang et al., 2019; Zhang et al., 2019). Studies demonstrate significant declines in plant community diversity, height, coverage, and biomass with increasing degradation severity (Li et al., 2025; Liu et al., 2025). Notably, high-quality forage species (grasses, legumes, and sedges) progressively decrease in biomass and are gradually replaced by toxic forbs (Peng et al., 2020; Wang et al., 2014). Degradation also alters soil structure and environmental conditions through changes in abiotic properties, negatively impacting aboveground vegetation (Han et al., 2020). For example, increased soil compaction from reduced moisture and soil salinization due to elevated pH can severely hinder plant nutrient uptake efficiency (Chen et al., 2023; Jing et al., 2015). Severe degradation can cause losses of 42% in soil organic carbon and 33% in total nitrogen content (Breidenbach et al., 2022). Consequently, alpine grassland degradation diminishes multiple ecosystem functions, ultimately compromising ecosystem multifunctionality (Wang et al., 2019; Xu et al., 2021).

The Qinghai Lake Basin is a critical ecological barrier in the northeastern Qinghai-Tibetan Plateau, essential for maintaining the region’s ecological security (Zhang et al., 2022). Since the 1950s, its alpine grasslands have undergone widespread degradation due to anthropogenic pressures (overgrazing, overcultivation, mining) and natural drivers (climate change, rodent/insect infestations) (Zhou et al., 2023). Although grassland protection measures (e.g., fencing, grazing bans, rotational grazing, replanting) implemented since 2000 have slowed degradation (Zhu et al., 2023), it remains a major ecological concern. This study employed a space-for-time substitution approach to investigate plant communities and soil characteristics across alpine steppe with varying degradation gradients (non-degraded, slightly degraded, moderately degraded, and severely degraded alpine steppe) in the Qinghai Lake Basin. To explore the effects of plant community diversity and dominance of dominant species on ecosystem multifunctionality in the context of alpine steppe degradation, we propose the following hypotheses: (1) Across different degradation gradients in alpine steppe, the relationships among plant community diversity, dominance of dominant species, and ecosystem multifunctionality are not simply linear (Baert et al., 2017); (2) During the degradation process, dominant species exert a controlling influence on resource allocation and micro-environment formation through their exceptional functional traits (such as high biomass and strong resource acquisition ability) due to strong environmental filtering (Grime, 1998; Hou et al., 2023). Thus, dominance of dominant species will be the key biotic driver of changes in ecosystem multifunctionality. The findings of this study will elucidate the non-linear nature of the diversity–ecosystem functioning relationships across degradation gradients in alpine steppe and clarify the central role of dominant species in maintaining ecosystem functions in degraded alpine steppe. This will not only deepen the mechanistic understanding of alpine steppe degradation but also provide a critical theoretical basis for ecological restoration practices focused on the conservation of key species.

## 2 Materials and methods

### 2.1 Study area

**Figure 1:**
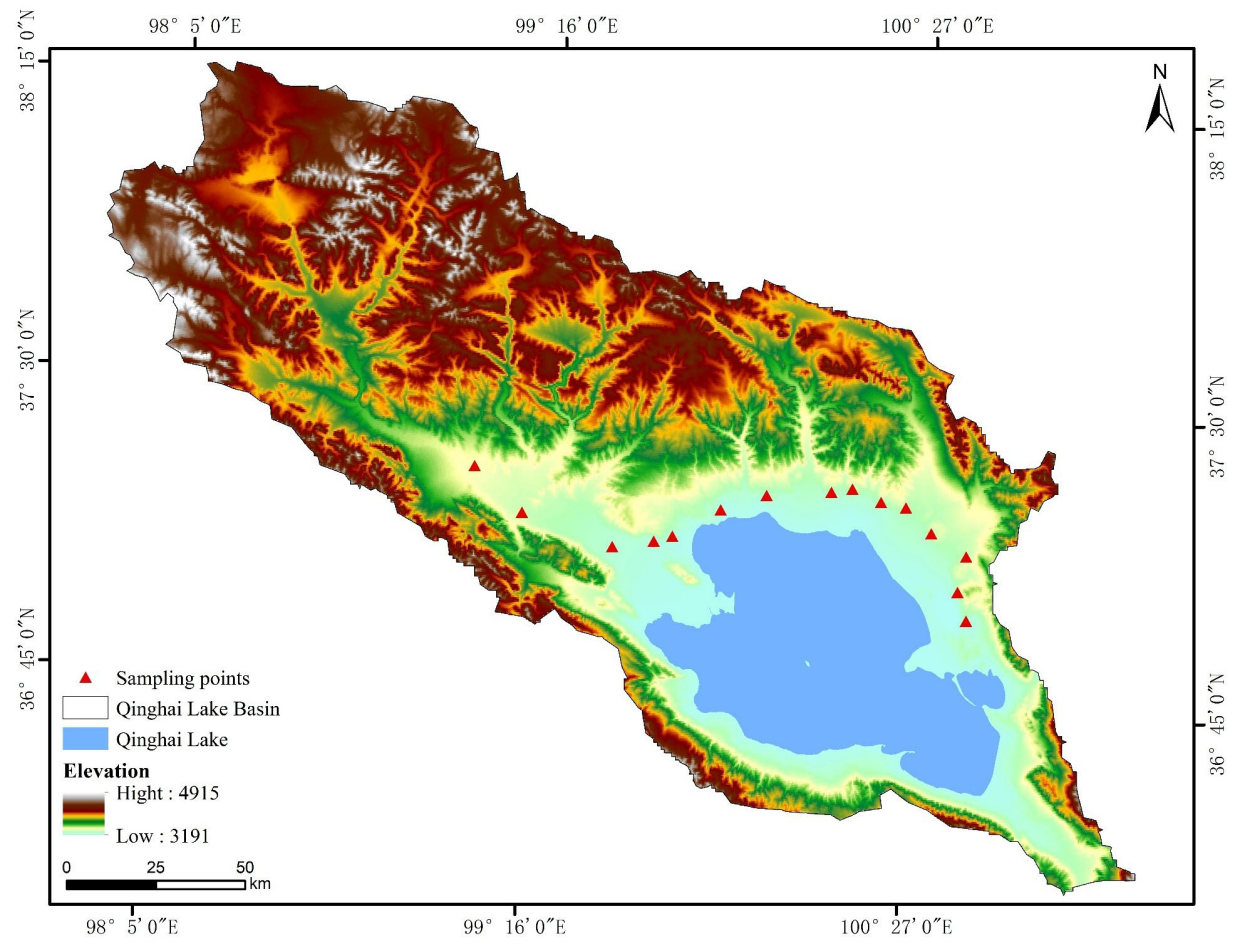
Location map of sampling points at the alpine steppe in Qinghai Lake Basin.

The Qinghai Lake Basin is situated in the northeastern region of the Qinghai-Tibet Plateau (latitude: 36°15’–38°20’N; longitude: 97°50’–101°20’E), at an elevation ranging from 3,194 to 5,291 meters (**Figure 1**). This basin exhibits a typical plateau continental climate, characterized by intense solar radiation, arid conditions, and low temperatures. The region experiences an annual sunshine duration of approximately 2,900 h, with average temperatures fluctuating between -0.1°C and 0.4°C. The mean annual precipitation is recorded between 291 and 579 mm, while the average annual evaporation ranges from 1,300 to 2,000 mm. The dominant species in the non-degraded alpine steppe is *Stipa purpurea* (relative important value: 0.45), and the common companion species are *Poa pratensis* (relative important value: 0.11), *Agropyron cristatum* (relative important value: 0.07), and *Leymus secalinus* (relative important value: 0.08). Additionally, the region supports a diverse array of forb species, including *Aster altaicus*, P*edicularis kansuensis*, *Bupleurum triradiatum*, and *Sibbaldianthe bifurca*, among others. The basin is characterized as an ecologically fragile area, marked by harsh environmental conditions, a brief plant growth cycle, and limited capacity for self-recovery following grassland degradation. The grasslands within this basin serve as winter pastures for Tibetan sheep, with grazing activities occurring from October through April (Wang et al., 2023b; Xiao et al., 2020).

### 2.2 Field sampling design

In this study, we investigated alpine steppe across four degradation gradients: non-degraded alpine steppe (ND), slightly degraded alpine steppe (LD), moderately degraded alpine steppe (MD), and severely degraded alpine steppe (SD). The classification of these degradation gradients was based on the local standard of Qinghai Province (DB63/T981-2011) (**Supplementary Table S1**). In accordance with the aforementioned standards, this classification is based on the dominant species (ND: grass, LD: grass + forb, MD: forb, and SD: toxic forb), the percentage of grass coverage (ND: >30%, LD: 20%-30%, MD: 10%-20%, and SD: <10%), and the proportion of herbage biomass (ND: >70%, LD: 50%-70%, MD: 30%-50%, and SD: <30%) (**Supplementary Table S1**). In selecting research sites for alpine steppes at various degradation levels, we not only adhered to these established criteria but also incorporated the expertise of seasoned researchers in grassland investigations to guide field assessments.

A total of 15 study sites were examined in the Qinghai Lake basin from August to September in 2023 and 2024. Each site encompassed areas representing all four degradation gradients. To minimize the influence of spatial distance, the distance between different degradation gradients was maintained within 2 km. Within each degraded alpine steppe, we established three distinct areas for the purpose of sample collection and plant community surveys. This approach was employed to ensure that the samples and surveys accurately represented the ecological characteristics of degraded alpine steppe.

### 2.3 Plant community survey and soil sampling

Plant community surveys were conducted using 1×1 m quadrats to record species composition, natural height, and species coverage. Aboveground biomass was measured by harvesting all plants within a nested 50×50 cm quadrat (maintaining 2 cm stubble height). Harvested plants were sorted into four functional groups (grasses, legumes, sedges, and forbs), oven-dried at 65°C for 48 hours, and weighed to determine dry biomass. Total aboveground biomass was calculated as the sum of all functional groups. In the alpine steppe along a degradation gradient, we examined six plant communities and collected nine aboveground biomass samples per community. Community structure was characterized using mean values of species richness, height, coverage, functional group biomass, and total aboveground biomass for each degradation level. Belowground biomass was sampled from three soil depths (0-10, 10-20, and 20–30 cm) using an 8-cm diameter root drill. After washing with running water, active roots were selected based on color and elasticity, oven-dried at 65°C to constant weight, and weighed.

Soil samples were collected using a soil drill with a diameter of 5 cm, stratified into layers (0–10 cm, 10–20 cm, 20–30 cm) within each biomass harvesting quadrat. Samples from the same soil layer were combined into a single composite sample. Each composite soil sample was divided into two portions: one portion was stored at 4°C for the analysis of ammonium nitrogen, nitrate nitrogen, and moisture content, while the other portion was air-dried away from direct sunlight for the determination of additional soil indexes (total nitrogen, total phosphorus, available phosphorus, organic carbon, and pH).

### 2.4 Determination of soil physicochemical properties

The air-dried soil sample is ground after removing roots, gravel and other debris, and then the soil is screened with a 60-mesh screen for the determination of soil physicochemical properties. A pH meter was used to determine soil pH (soil: water = 1: 2.5) (PB-10, Germany). Soil organic carbon (SOC) was determined by external heating method with potassium dichromate. The content of total nitrogen (TN) in soil was determined by Kjeldahl method. Determination of total phosphorus (TP) and available phosphorus (AP) in soil by Mo-Sb colorimetric method. The ammonium nitrogen and nitrate nitrogen in fresh soil samples were extracted by KCl solution (2 mol/L), and the content of ammonium nitrogen and nitrate nitrogen in the extracted solution was determined using a discrete multichemistry analyzer (Seal AQ1, Germany). Drying constant weight method was used to evaluate the soil moisture (SM) (Bao, 2005).

### 2.5 Species diversity and ecosystem multifunctionality index calculation

In this study, the dominance of dominant species was the relative important value (RIV) of *Stipa purpurea.* Relative important value, species richness, Shannon-Wiener index, Simpson index and Pielou evenness index were used to characterize plant community diversity. The calculation formula is as follows (Mori et al., 1983; Pielou, 1966; Shannon, 1948; Simpson, 1949).


(1) RIV=(RH+RC)/2

(2) S=n

(3) $$H=-\sum P_{i}lnP_{i}$$

(4) $$D=1-\sum P_{i}^{2}$$

(5) E=H/lnS

Where RIV is the relative important value of each species, RH is the relative height of each species, RC is the relative coverage of each species, S is the species richness, n is the number of species in the quadrat, H is the Shannon-Wiener index, P*i* is the relative importance of *i* species, D is Simpson index, E is the Pielou evenness index.

Ecosystem multifunctionality (EMF) indicators were chosen for integration based on existing frameworks (Hector and Bagchi, 2007; Maestre et al., 2012). The EMFs were derived through factor analysis, utilizing 11 indicators: aboveground biomass, belowground biomass, plant height, vegetation cover, soil total nitrogen, total phosphorus, available nitrogen (nitrate nitrogen and ammonium nitrogen), available phosphorus, organic matter, soil moisture and pH (Shu et al., 2023). The selected indicators reflect the primary productivity and nutrient cycling processes of alpine steppe ecosystems.

The indicators used to calculate ecosystem multifunctionality were first standardized as much as possible, using the Min-Max standardization method. The standardization formula is as follows.


(6) $$SV=(X-\;X_{min})/(X_{max}-\;X_{min})$$

Where SV is the standardized value for each indicator, X is the measured value, X_min_ is the minimum value and X_max_ is the maximum value.

The standardized ecosystem functional indicators were subjected to Bartlett’s Test of Sphericity. The related eigenvalues, factor loadings and percent of variance explained were obtained through factor analysis, and the ecosystem multifunctionality index was calculated by the following formula.


(7) $$EMF=\;\sum a_{i}z_{i}$$

(8) $$z_{i}=\;\sum w_{ij}x_{ij}$$

Where, a_i_ is the percent of variance explained of each factor, z_i_ is the factor score, w_ij_ is the factor score coefficient of the ground i variable at the j factor, and x_ij_ is the standardized value of the i variable at the j factor.

### 2.6 Statistical analyses

Prior to data analysis, the Kolmogorov-Smirnov and Bartlett tests were employed to assess the normality and homogeneity of variance, respectively. Data not adhering to a normal distribution were transformed using a logarithmic function, after which further analyses were conducted. A one-way ANOVA was utilized to evaluate the effects of varying degrees of degradation on soil physicochemical characters, plant diversity, biomass, plant community height and coverage, factor scores and ecosystem multifunctionality index. The study sites and degradation gradients were treated as fixed factors, and Tukey’s HSD test was applied to determine significant differences among degradation gradients at a significance level of *P* < 0.05. A general linear model (GLM) was employed to examine the relationships between plant community diversity and the dominance of dominant species and ecosystem multifunctionality in different degraded gradients of alpine steppe. Model selection was based on the Akaike Information Criterion (AIC) and the coefficient of determination (*R*²). A lower AIC value and a higher *R*² value indicate a better-fitting model. Linear mixed models (LMM) were employed to investigate the effects of plant community diversity and the dominance of dominant species on ecosystem multifunctionality during the degradation of alpine steppe. The absolute values of the fixed-effect estimates were interpreted as the magnitude of influence. Marginal R² and conditional R² were manually computed using calculation formulas referenced from the work of Nakagawa et al. (2017). The specific calculation formulas are provided below.


(9) $$R_{m}^{2}=\frac{\sigma_{f}^{2}}{\sigma_{f}^{2}+\sigma_{l}^{2}+\sigma_{e}^{2}}$$

(10) $$R_{c}^{2}=\frac{\sigma_{f}^{2}+\sigma_{l}^{2}}{\sigma_{f}^{2}+\sigma_{l}^{2}+\sigma_{e}^{2}}$$

Where $R_{m}^{2}$ denotes the marginal R², $\sigma_{f}^{2}$ represents the variance attributed to fixed effects, $\sigma_{l}^{2}\;$ corresponds to the variance component of random effects, and $\sigma_{e}^{2}\;$ signifies the residual variance, $R_{c}^{2}$ denotes the conditional R².

## 3 Results and analysis

### 3.1 Ecosystem multifunctionality in alpine steppe with different degradation gradients

**Figure 2:**
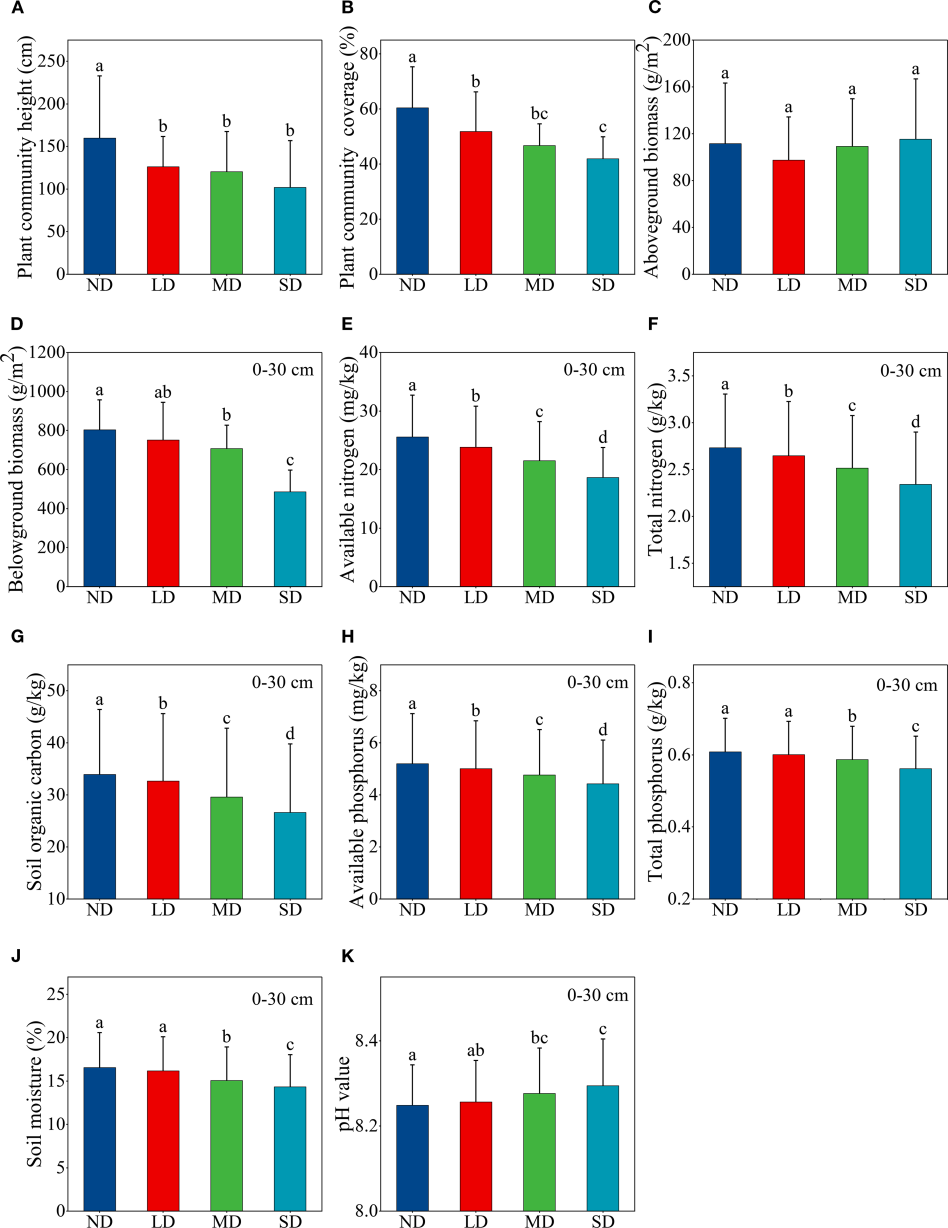
Changes in aboveground and belowground ecosystem functions along an alpine steppe degradation gradient. **(A)** plant community height; **(B)** plant community coverage; **(C)** aboveground biomass; **(D)** belowground biomass; **(E)** available nitrogen; **(F)** total nitrogen; **(G)** soil organic carbon; **(H)** available phosphorus; **(I)** total phosphorus; **(J)** soil moisture; **(K)** pH value. ND: non-degraded alpine steppe; LD: slightly degraded alpine steppe; MD: moderately degraded alpine steppe; SD: severely degraded alpine steppe. Different lowercase letters signify significant differences in the functional indicators among degradation gradients (P < 0.05).

The height and coverage of plant community in alpine steppe decreased significantly with increasing levels of degradation (**Figures 2A, B**). In comparison to non-degraded alpine steppe, those that were moderately and severely degraded demonstrated a significant reduction in belowground biomass (**Figure 2D**). Conversely, no significant variation in aboveground biomass was observed across the degradation gradient (**Figure 2C**). Degradation of alpine steppe led to decline in soil nutrients and soil moisture, while significantly increasing soil pH (**Figures 2E–K**). The contents of soil total nitrogen, available nitrogen, organic carbon, and available phosphorus decreased significantly as a result of alpine steppe degradation (**Figures 2E–H**). Moreover, soil total phosphorus content and moisture were significantly lower, and soil pH was significantly higher in moderately and severely degraded alpine steppe compared to the non-degraded (**Figures 2I–K**).

**Figure 3:**
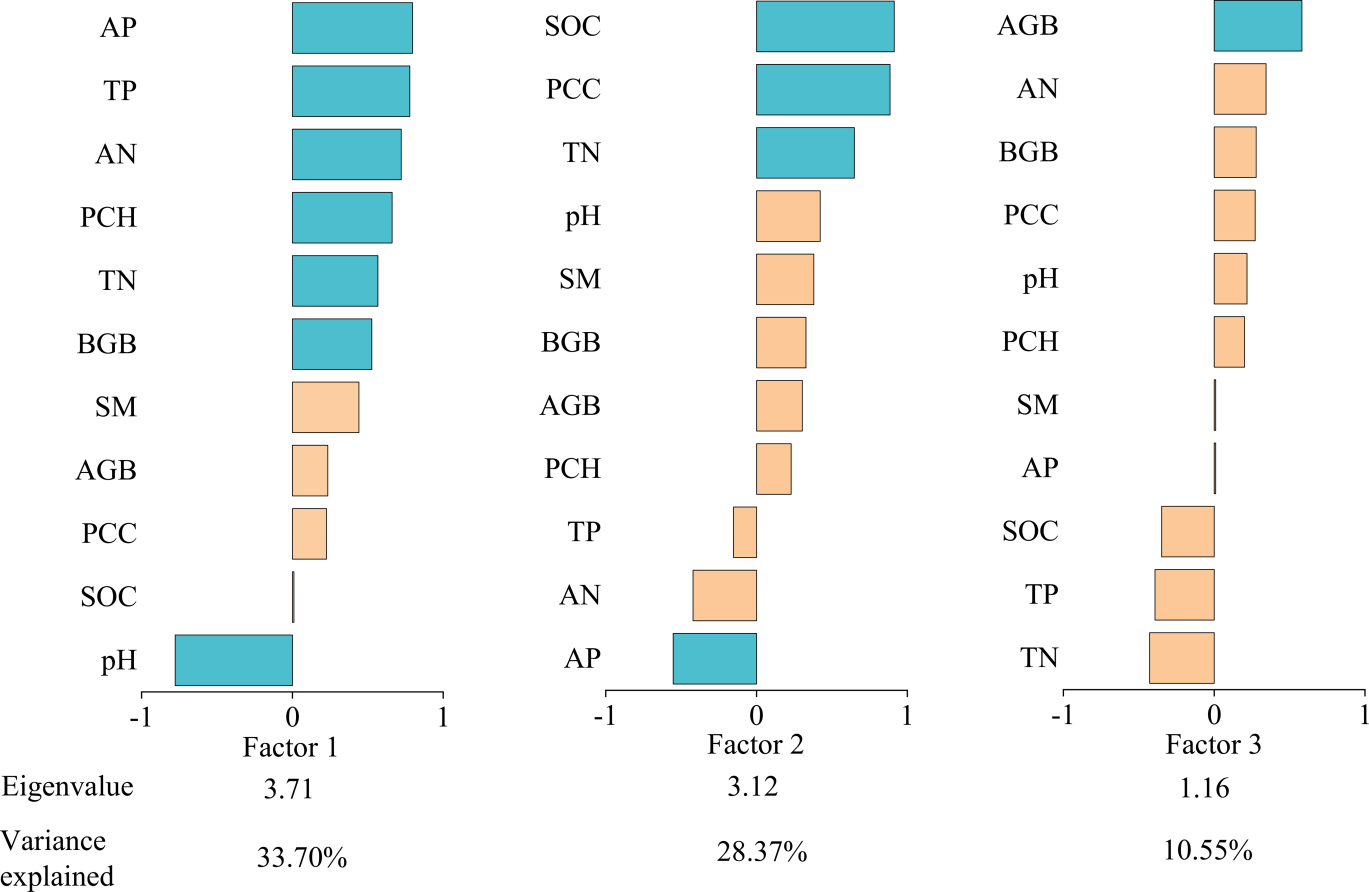
The eigenvalues, factor loadings and percent of variance explained. AP, available phosphorus; TP, total phosphorus; AN, available nitrogen; PCH, plant community height; TN, total nitrogen; BGB, belowground biomass; SM, soil moisture; AGB, aboveground biomass; PCC, plant community coverage; SOC, soil organic carbon.

We performed multivariate downscaling and extracted factors from 11 soil and vegetation functional indicators. We calculated eigenvalues, factor loadings, and variance percentages for each indicator relative to common factors (**Figure 3**). Three factors with eigenvalues >1 were identified, collectively explaining 72.62% of total variance (cumulative eigenvalue=7.99). Factor 1 showed strong loadings from AP, TP, AN, PCH, TN, BGB, and pH; Factor 2 from SOC, PCC, TN, and AP; and Factor 3 primarily from AGB. Each factor represents a set of synergistically varying (redundant) functional components, while the relationships between different factors represent the primary trade-offs within the system. The functions within Factor 1 and Factor 2 exhibit a high degree of synergism. The functions loading on Factor 1 reflect the capacity and availability of ecosystem nutrient pools, as well as plant resource acquisition. The functions within Factor 2 are closely interrelated and collectively maintain the nutrient cycling and physical structure of the system. Factor 3 is dominated by a single function, indicating that aboveground biomass represents a relatively independent functional strategy within our ecosystem. While Factor 1 and Factor 2 share common indicators (such as TN and AP), suggesting an association between them, their distinct constituent elements (pH and BGB in Factor 1, versus SOC and PPC in Factor 2) indicate that they represent more nuanced strategic differentiation within the broader framework of system maintenance functions. We calculated ecosystem multifunctionality scores using factor scores across degradation gradients (**Table 1**). These scores decreased progressively with degradation severity, indicating significant deterioration in alpine steppe ecosystem function.

**Table 1:** Factor scores and ecosystem multifunctionality index of plant community in degraded alpine steppe.

Degradation gradients	Factor score			Ecosystem multifunctionality index
	Factor 1	Factor 2	Factor 3	
ND	0.213 a	0.251 a	0.675 a	21.397 a
LD	0.153 a	0.181 a	0.041 b	10.720 b
MD	-0.040 b	-0.090 b	-0.088 bc	-4.826 c
SD	-0.326 c	-0.341 c	-0.628 c	-27.291 d

ND, non-degraded alpine steppe; LD, slightly degraded alpine steppe; MD, moderately degraded alpine steppe; SD, severely degraded alpine steppe. Different lowercase letters signify significant differences in the factor scores and ecosystem multifunctionality index among degradation gradients (*P* < 0.05).

### 3.2 Relationship between plant community diversity and dominance of dominant species, and ecosystem multifunctionality in alpine steppe with different degradation gradients

**Figure 4:**
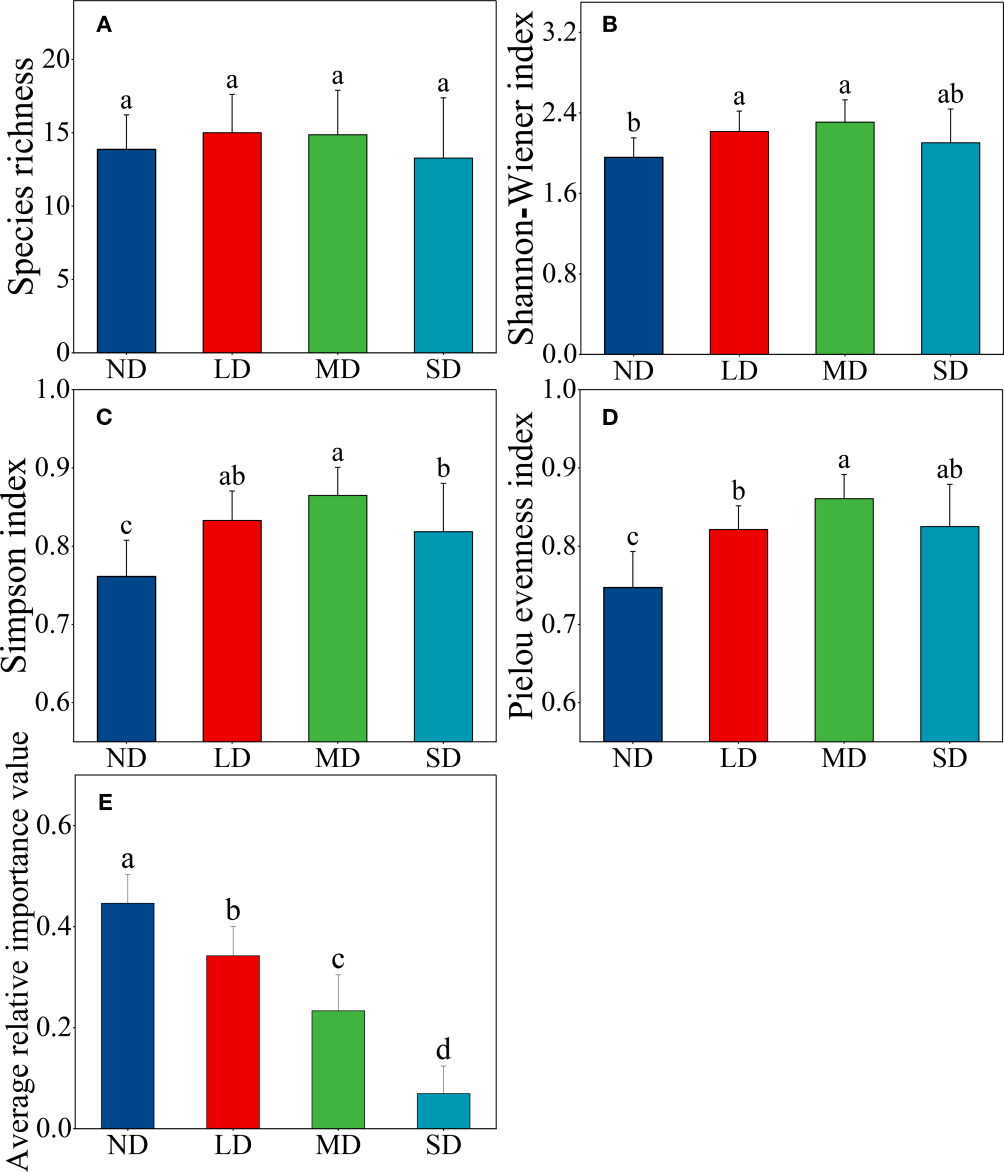
Changes in plant community diversity **(A)** species richness, **(B)** Shannon-Wiener index, **(C)** Simpson index, and **(D)** Pielou evenness index) and the dominance of dominant species **(E)** average relative importance value) along an alpine steppe degradation gradient. ND: non-degraded alpine steppe; LD: slightly degraded alpine steppe; MD: moderately degraded alpine steppe; SD: severely degraded alpine steppe. Different lowercase letters signify significant differences in the functional indicators among degradation gradients (*P* < 0.05). Relative importance value indicates the relative dominance of dominant species in plant communities.

In response to the degradation of alpine steppe, changes in plant community diversity exhibited a unimodal pattern, with the highest diversity observed at moderate levels of degradation (**Figures 4A–D**). Concurrently, the dominance of dominant species (Equation 1) experienced a marked decline (**Figure 4E**). The degradation of alpine grasslands led to a significant increase in both the Simpson index and Pielou evenness index of plant communities (**Figures 4C, D**). Furthermore, slight to moderate degradation resulted in a notable enhancement of the Shannon-Wiener index compared to the non-degraded alpine steppe (**Figure 4B**).

**Figure 5:**
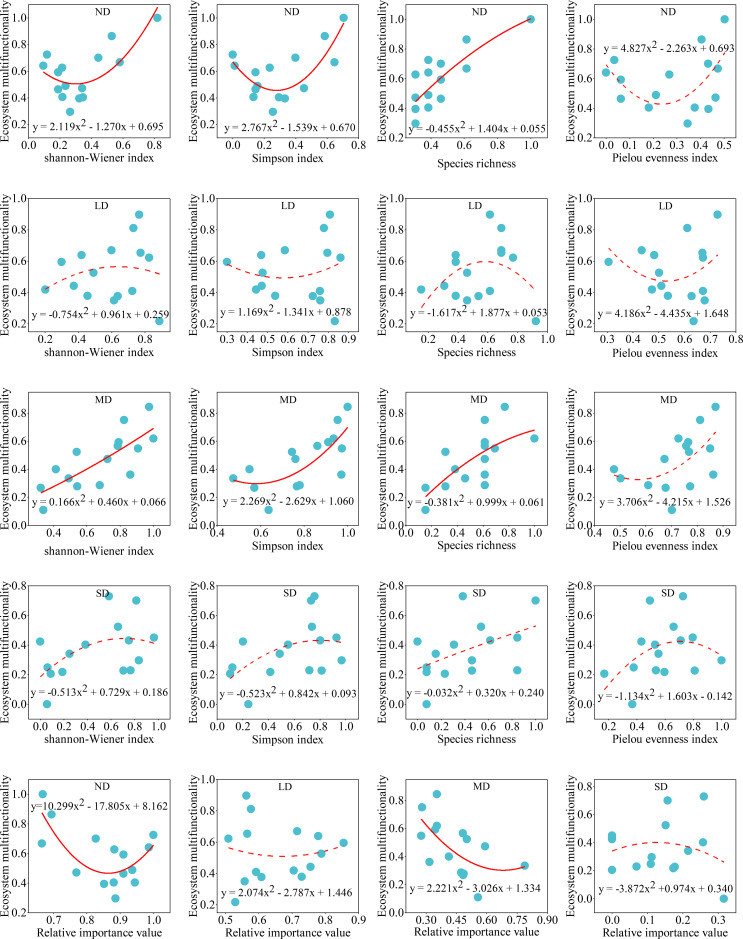
Relationship between ecosystem multifunctionality and plant community diversity (species richness, Shannon-Wiener index, Simpson index, and Pielou evenness index) and dominance of dominant species in alpine steppe with different degradation gradients. ND: non-degraded alpine steppe; LD: slightly degraded alpine steppe; MD: moderately degraded alpine steppe; SD: severely degraded alpine steppe. Relative importance value indicates the relative dominance of dominant species in plant communities. Solid line: *P* < 0.05; dashed lines: *P* > 0.05.

Regression analysis revealed that alpine steppe ecosystem multifunctionality followed a binomial rather than linear relationship with plant diversity (Equations 2–5) and dominance of dominant species across degradation gradients (**Figure 5**). The binomial model showed superior fit, with lower AIC and higher *R*² values compared to the linear model (**Supplementary Table S2**). In non-degraded and moderately degraded alpine steppe, ecosystem multifunctionality responded significantly to Shannon-Wiener index, Simpson index, and species richness (*P*<0.05), but not to Pielou evenness index (*P* > 0.05) (**Supplementary Table S2**). Similarly, ecosystem multifunctionality (Equations 6–8) significantly responded to dominance of dominant species in non-degraded and moderately degraded alpine steppe. However, the linear relationship between dominance of the dominant species and ecosystem multifunctionality was significant (*P* < 0.05) in moderately degraded alpine steppe.

### 3.3 Relationship between ecosystem multifunctionality and plant community diversity and dominance of dominant species during alpine steppe degradation processes

**Figure 6:**
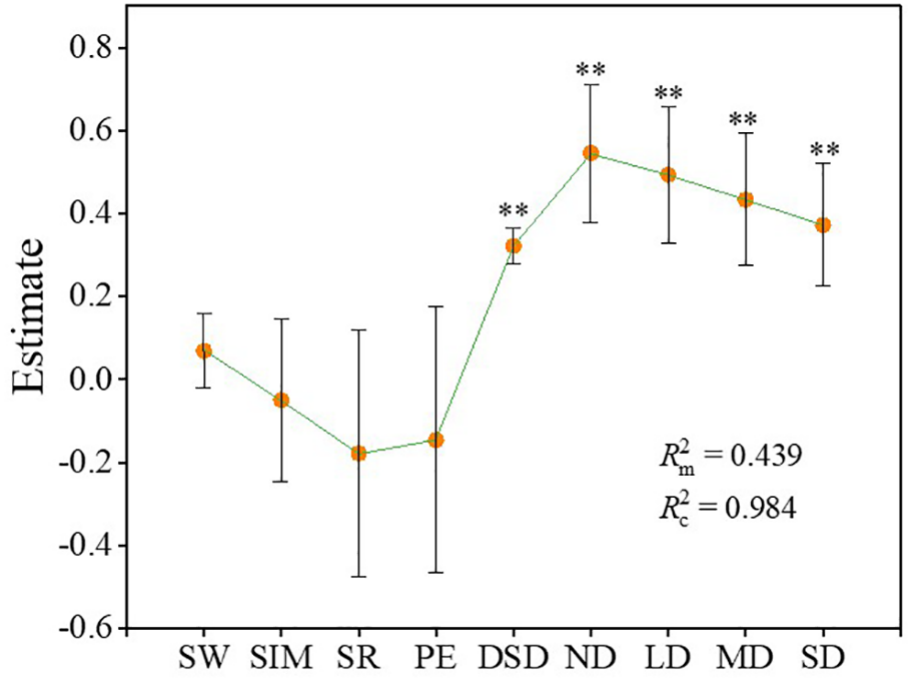
Linear mixed models of the effects of plant diversity (species richness, Shannon-Wiener index, Simpson index, and Pielou evenness index) and dominance of dominant species on ecosystem multifunctionality. The error bar represents the confidence interval (95% CI). $R_{m}^{2}\;$ is Marginal R^2^, $R_{c}^{2}$ is Conditional R^2^. SW, Shannon-Wiener index; SIM, Simpson index; SR, species richness; PE, Pielou evenness index; DSD, dominance of the dominant species. ND, non-degraded alpine steppe; LD, slightly degraded alpine steppe; MD, moderately degraded alpine steppe; SD, severely degraded alpine steppe. **: *P* < 0.01.

During the degradation process of alpine steppe, the linear mixed model results demonstrated that both the degradation gradient and the dominance of dominant species significantly influenced ecosystem multifunctionality. Moreover, the degradation gradient exerted a stronger influence on ecosystem multifunctionality than the dominance of dominant species (absolute values of estimate for ND, LD, MD, SD: 0.57, 0.52, 0.46, 0.40; absolute value of estimate for dominance of dominant species: 0.32) (**Figure 6**) (**Supplementary Table S3**). Both fixed and random effects (Equations 9, 10) collectively accounted for approximately 98.4% of the variation in ecosystem multifunctionality. Specifically, the fixed effects (plant community diversity, dominance of dominant species, and degradation gradient) and the random effects (site-specific differences) explained approximately 43.9% and 54.5% of the variation in ecosystem multifunctionality during the degradation process of alpine steppe, respectively (**Figure 6**).

## 4 Discussion

### 4.1 Effects of alpine grassland degradation on ecosystem characteristics

Reduced plant community biomass, height, and coverage are key indicators of grassland degradation (Tang et al., 2014; Zhou et al., 2014). In this study, the degradation of alpine grasslands significantly reduced plant community height and coverage (**Figures 1A, B**). In the Qinghai Lake basin, alpine steppe degradation is primarily caused by human activities, particularly overgrazing (Gao and Carmel, 2020; Wang et al., 2006). Plant community height and coverage in alpine grasslands decreased as degradation intensified due to grazing pressure (Luo et al., 2018; Teng et al., 2020). Furthermore, the results of this study indicated that aboveground biomass of the plant community did not show significant changes with progressive degradation of the alpine steppe (**Figure 2C**). Wang et al. (2014) reported declining biomass in grasses, sedges, and legumes along a degradation gradient, contrasting with increased forb biomass. In this study, grass biomass significantly declined with alpine steppe degradation, whereas forb biomass increased (**Supplementary Figure S1**). Alpine steppe ecosystems are inherently fragile due to their low vegetation coverage, biomass, and biodiversity compared to alpine meadows, as well as their vulnerability to cold, arid climates and external disturbances (Hao et al., 2020, 2021). Consequently, non-degraded alpine steppe—primarily composed of graminoids—exhibit low total biomass. During degradation, the loss of grass biomass is often compensated by increased forb biomass. This compensatory mechanism stabilizes total aboveground biomass across degradation gradients, helping maintain primary productivity in grassland ecosystems. Although forb-like monocots contribute substantial biomass, their scattered distribution results in reduced plant community coverage as degradation intensifies. Nevertheless, total aboveground biomass remains relatively stable due to the structural characteristics of forb species.

This study showed that although the degradation of alpine steppe reduced the belowground biomass of plant communities (0–30 cm) (**Figure 2D**), the belowground biomass of alpine steppe plant communities was mainly concentrated in the 0–10 cm layer (**Supplementary Figure S2**). Non-degraded alpine steppe communities, dominated by graminoid with fibrous root systems concentrated in the upper soil layer, exhibits higher surface root biomass. However, as degradation progressed, graminoid decline led to decreased belowground biomass in this zone. Livestock trampling exacerbates this effect by compacting soil and impairing both photosynthesis and root growth (Lu et al., 2017). Concurrently, degradation-induced losses of surface soil nutrients and moisture compel plants to develop deeper root systems (Li et al., 2006). Notably, invading forb species do not compensate for surface belowground biomass loss, as their taproot-dominated systems grow vertically with dispersed distributions to reduce competition (Li et al., 2018). This shift was evidenced by the increased proportion of belowground biomass at 10–20 cm depths in severely degraded areas (**Supplementary Figure S2D**), suggesting opportunistic species adopt deep-foraging strategies to survive resource-limited conditions.

Grassland degradation, marked by vegetation and soil deterioration, progresses through complex interactions between these components (Li et al., 2022; Liu et al., 2024). Our results showed significantly increased soil pH and decreased moisture in moderately/severely degraded alpine steppe (**Figures 2J, K**). Overgrazing reduces vegetation coverage, exacerbating wind/water erosion and exposing soil to greater solar radiation (Gonzalez and Ghermandi, 2021). This enhances evapotranspiration (ET) in these naturally high-ET soils, lowering moisture while increasing salinity and pH (Wang and Wesche, 2016). Degradation progressively reduced soil organic carbon (SOC), total nitrogen (TN), and total phosphorus (TP) (**Figures 2F, G, I**). These nutrients primarily originate from plant litter and root exudates (Lei et al., 2023; Lu et al., 2023; Shen et al., 2020), both of which decline with degradation. Reduced belowground biomass (**Figure 2D**) limits root exudates, while livestock consumption of aboveground biomass decreases litter input (Gonzalez and Ghermandi, 2021; Wu et al., 2020). Although forb invasion increases aboveground biomass, it cannot compensate for nutrient losses. Livestock further deplete nutrients by consuming roots and nutrient-rich soils. Moderate-to-severe degradation decreased available nitrogen and phosphorus (**Figures 2E, H**) through: (1) increased erosion of exposed soil, and (2) reduced microbial mineralization due to moisture loss (Chen et al., 2013; Kraamwinkel et al., 2021). This dual mechanism creates a nutrient-depletion feedback loop that accelerates degradation.

Grassland degradation, driven by climate change and anthropogenic activities, significantly alters plant community structure and composition, thereby affecting vegetation diversity (Wang et al., 2019; Yang and Sun, 2021). This study reveals a unimodal relationship between plant diversity and alpine steppe degradation, with peak diversity occurring at moderate degradation levels (**Figure 4**). This pattern supports the *intermediate disturbance hypothesis*, which suggests that moderate disturbance enhances species diversity by reducing the dominance of certain species, thereby allowing others to access resources more effectively (Fox, 1979; Gao and Carmel, 2020). Overgrazing has drastically reduced the dominance of *Stipa purpurea*, the previously dominant species, creating niche opportunities for forb species (Sun et al., 2021; Yang et al., 2020). Consequently, species richness and evenness increased as grass dominance declined. However, when degradation progresses to severe levels, soil nutrients and water content (0–30 cm depth) diminish significantly, and allelopathic effects from toxic forbs further reduce diversity by excluding less competitive species (Wu et al., 2011). Teng et al. (2020) observed that plant diversity follows a hump-shaped trend—initially rising with moderate degradation before declining under severe degradation. Moreover, the limited species pool in alpine steppes restricts the introduction of new species during community transitions, keeping species richness relatively stable despite degradation (**Figure 4A**). Future research should investigate the local species pool, community assembly processes, and species migration mechanisms to better understand how plant diversity responds to varying degradation intensities in the Qinghai Lake basin’s alpine steppe.

### 4.2 Relationship between plant community diversity, the dominance of dominant species, and ecosystem multifunctionality in the context of alpine steppe degradation

Plant community diversity typically enhances ecosystem functioning through species complementarity and environmental selection effects (Loreau and Hector, 2001; Xu et al., 2024). Our study revealed a nonlinear (binomial) relationship between plant diversity and ecosystem multifunctionality across degradation gradients in alpine steppe (**Figure 5**). While greater diversity generally improves productivity, nutrient cycling, and overall ecosystem functioning (Cappelli et al., 2022; Hautier et al., 2018), this relationship appears mediated by dominant species presence and becomes nonlinear over time (Baert et al., 2017; Li et al., 2010). In this study, the response of ecosystem multifunctionality in non-degraded and moderately degraded alpine steppe to plant community diversity was found to be significant (**Figure 5**) (**Supplementary Table S2**). Grassland degradation may lead to either an increase or decrease in plant diversity (Wu et al., 2024; Yang et al., 2020), but the index of ecosystem multifunctionality exhibited a significant decline with grassland degradation, particularly concerning plant community coverage, height, and soil physicochemical properties (**Figure 2**). Consequently, alpine steppe degradation impacts the relationship between plant community diversity and ecosystem multifunctionality. Notably, ecosystem multifunctionality correlated significantly with Shannon-Wiener index, Simpson index, and species richness in non-degraded and moderately degraded alpine steppe (**Figure 5**) (**Supplementary Table S2**). This suggests alpine steppe multifunctionality depends more on species richness than distribution evenness.

In this study, the dominance of dominant species and ecosystem multifunctionality exhibited a binomial relationship in non-degraded and moderately degraded alpine steppe (**Supplementary Table S2**). This result is similar to the relationship between plant community diversity and ecosystem multifunctionality. Species dominance is an indicator of aggregation, and aggregation is the opposite of diversity (Grman et al., 2010; Sasaki and Lauenroth, 2011). The relationship between the dominance of dominant species and plant community diversity makes them similar to the relationship with ecosystem multifunctionality. However, during the degradation process of the alpine steppe, both plant community dominance and ecosystem multifunctionality decreased with increasing degradation intensity, demonstrating a synchronous trend (**Figure 4E**) (**Table 1**). Therefore, in the linear mixed model, the dominance of dominant species exerted a significant influence on the changes in ecosystem multifunctionality during the degradation of the alpine steppe (**Figure 6**). Dominant species often exhibit greater coverage, height, and biomass, serving as key factors in maintaining the structural stability of plant communities (He et al., 2019; Garnier et al., 2004). The mass ratio hypothesis proposes that ecosystem properties are largely determined by the functional traits of these dominant species within the community (Garnier et al., 2004; Grime, 1998). Therefore, the findings of this study are consistent with the mass ratio hypothesis. Furthermore, the linear mixed model indicated that both the degradation gradient and site-specific effects exerted significant influences on changes in ecosystem multifunctionality during alpine steppe degradation. This result demonstrates that shifts in plant community composition and soil properties induced by the degradation gradient, along with climatic and elevational factors associated with site effects, collectively mediate ecosystem multifunctionality.

Alpine steppe degradation alters dominant species composition, subsequently affecting ecosystem functioning through multiple pathways. Dominant species directly influence key ecosystem functions such as biomass production and nutrient cycling (Chen et al., 2023; Garnier et al., 2004). In degraded alpine steppe, overgrazing reduces dominant species abundance, decreasing plant height and coverage. This reduction (1) increases vulnerability to wind/water erosion and (2) enhances soil moisture evaporation through greater solar exposure. The decline of dominant species also reduces belowground biomass, limiting root exudates and litter inputs that maintain soil nutrients (Zhou et al., 2024). Furthermore, livestock removal of dominant species disturbs root zones and soil structure, accelerating sandification. These combined effects progressively impair the functional capacity of alpine steppe ecosystems.

Grassland degradation fundamentally alters plant community composition, marked by declining high-quality forage grasses and increasing forbs dominance (Li et al., 2015; Zhou et al., 2023). The expansion of forbs exacerbates the decline of ecosystem multifunctionality in grasslands, as invasive forbs fail to compensate for losses in belowground biomass or vegetation structure, thereby increasing ecosystem vulnerability. Furthermore, the loss of dominant species alters soil microbial communities (Chen et al., 2023), impairing nutrient cycling and potentially further influencing ecosystem multifunctionality. Furthermore, the loss of dominant species alters soil microbial communities (Chen et al., 2023), impairing nutrient cycling, which could subsequently influence ecosystem multifunctionality.

In conclusion, alpine steppe degradation reduces the dominance of dominant species, directly or indirectly affecting the multifunctionality of ecosystems. The decline of dominant species emerges as the principal driver of reduced ecosystem functioning during degradation. Therefore, restoring degraded alpine grasslands should first focus on: (1) reestablishing dominant species populations, and (2) improving soil conditions, before addressing plant community diversity enhancement.

## 5 Conclusions

Alpine steppe degradation in the Qinghai Lake Basin has significantly altered ecosystem characteristics and reduced multifunctionality. Our study revealed three key findings: (1) Plant community diversity followed a unimodal response to degradation, peaking at moderate levels while grass height, coverage, and biomass progressively declined. Forb biomass showed an inverse relationship, indicating a structural shift from graminoid to forb dominance. (2) Across different degradation gradients in alpine steppe, degradation weakened the relationship between diversity indices (Simpson index, Shannon-Wiener index, and species richness) and ecosystem multifunctionality. Multifunctionality correlated strongly with species richness and diversity indices (Shannon-Wiener index, Simpson index) but not with Pielou evenness index, demonstrating greater dependence on species richness than distribution evenness. (3) Results from the linear mixed model indicated that the dominance of dominant species exerted a significant effect on changes in ecosystem multifunctionality during the degradation of the alpine steppe, which is consistent with the mass ratio hypothesis. These findings suggest that restoration efforts should prioritize dominant species recovery to enhance ecosystem functioning in degraded alpine steppe.

## Data Availability

The original contributions presented in the study are included in the article/**Supplementary Material**. Further inquiries can be directed to the corresponding author.
