# Supplementary figures and images for: Alpine steppe degradation weakens ecosystem multifunctionality through the decline in climax dominant species on the Qinghai-Tibetan plateau
Source: Front Plant Sci. 2025 Oct 6;16:1650352. doi: 10.3389/fpls.2025.1650352 (PMC12535974; doi:10.3389/fpls.2025.1650352)

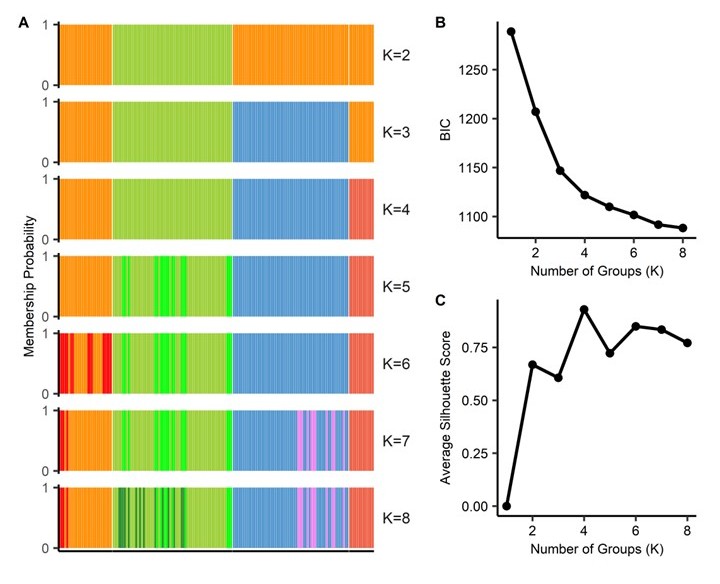

Supplement: Supplementary file 2 [file Image1.jpeg]

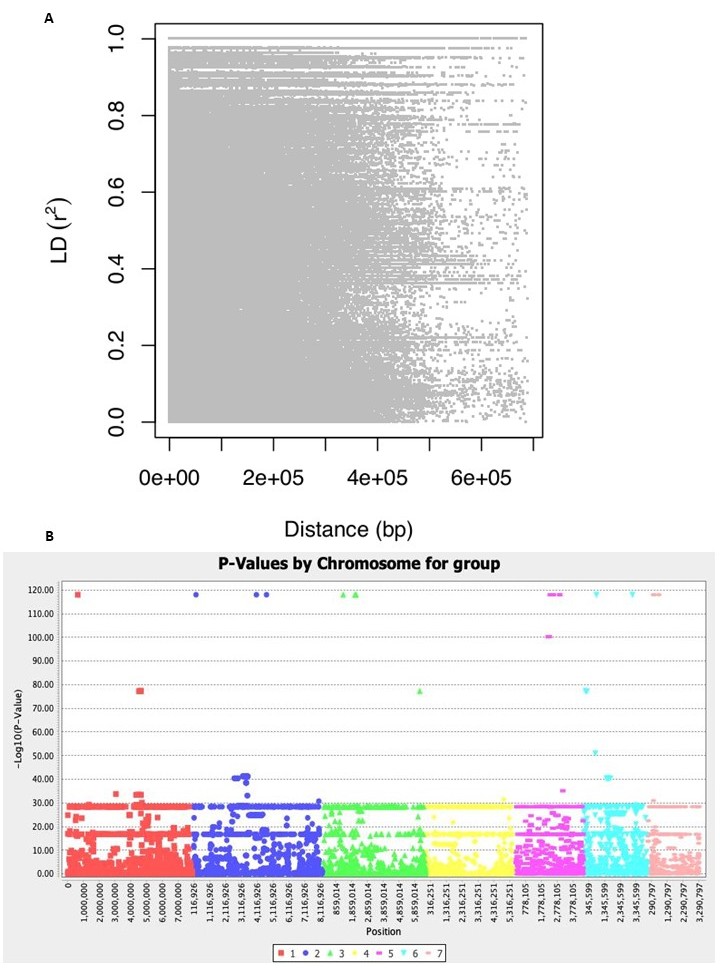

Supplement: Supplementary file 3 [file Image2.jpeg]

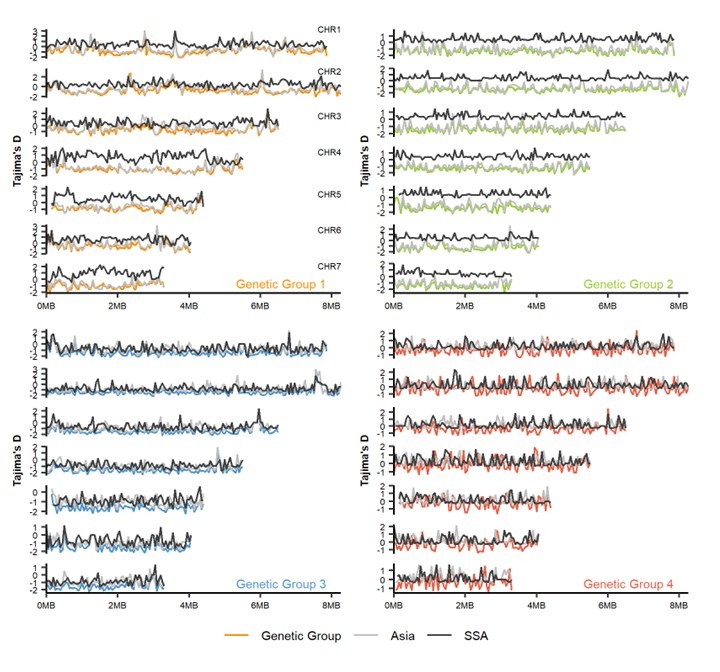

Supplement: Supplementary file 4 [file Image3.jpeg]

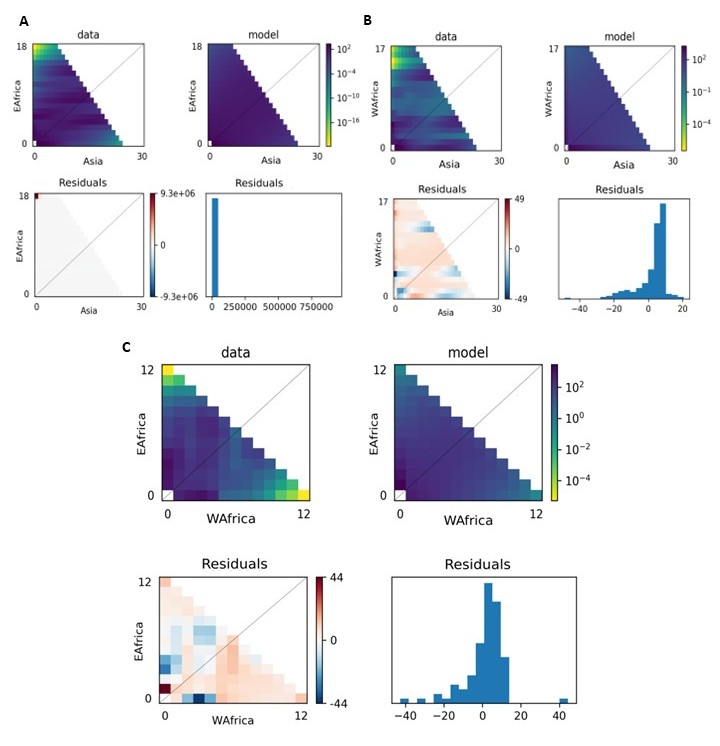

Supplement: Supplementary file 5 [file Image4.jpeg]

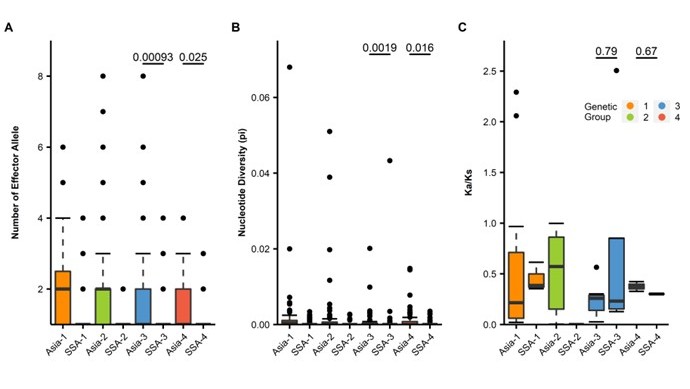

Supplement: Supplementary file 6 [file Image5.jpeg]

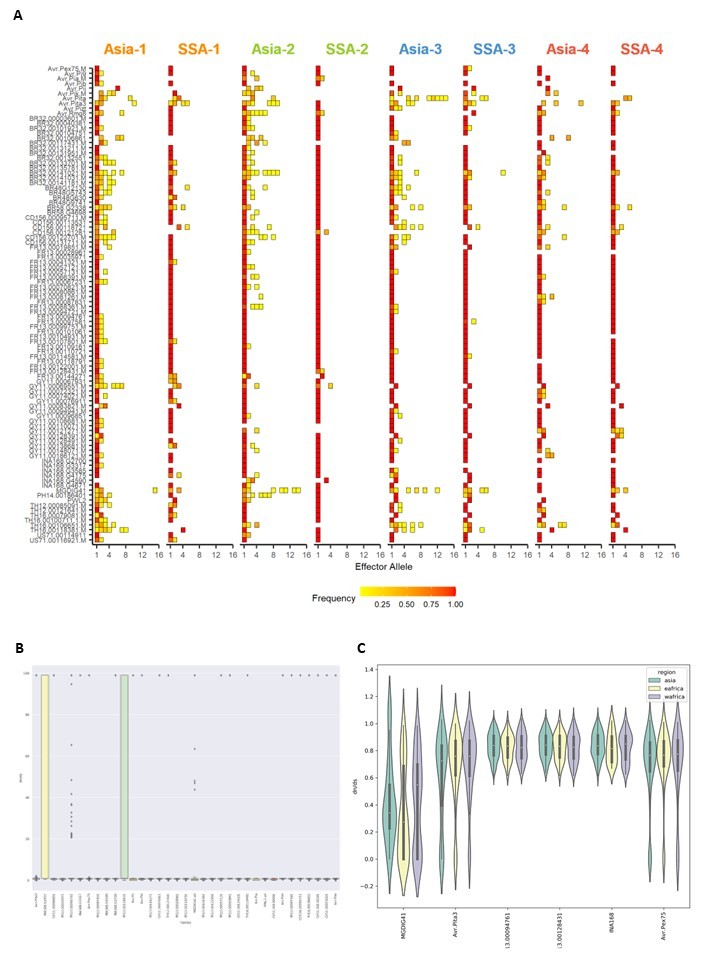

Supplement: Supplementary file 7 [file Image6.jpeg]
